# Isolated Abducens Nerve Palsy Following Spinal Anesthesia

**DOI:** 10.7759/cureus.41298

**Published:** 2023-07-03

**Authors:** Hing Siau Tiak, Mimiwati Zahari

**Affiliations:** 1 Ophthalmology, University Malaya Eye Research Centre, Kuala Lumpur, MYS

**Keywords:** spinal anesthesia, intracranial hypotension, sixth cranial nerve palsy, abducens nerve palsy, diplopia

## Abstract

A healthy 28-year-old lady, para 1, presented to the emergency department with persistent frontal headache, nausea, and vomiting following an emergency cesarean section four days ago. She experienced difficulties with six failed attempts of spinal anesthesia intrapartum before conversion to general anesthesia. A 25-gauge Whitacre needle was utilized for administering spinal anesthesia under a sitting position. The anesthetist noticed a loss of resistance upon needle insertion, but only a negligible amount of cerebrospinal fluid was obtained upon removing the stylet. The patient underwent an emergency cesarean section due to fetal distress, and she was not in labor during the attempts of spinal anesthesia. Otherwise, the cesarean section lasted for an hour and was uneventful. No intrapartum eclampsia or pre-eclampsia.

She was diagnosed with post-dural puncture headache, and her symptoms improved after receiving intravenous hydration, oral caffeine, and non-steroidal anti-inflammatory drug (NSAIDs). However, on the sixth day after the spinal anesthesia, she suddenly developed double vision. Examination showed bilateral visual acuity was measured at 6/7.5. No proptosis or ptosis was noted. The relative afferent pupillary defect was negative with no anisocoria. Both eyes were orthophoria with normal head posture. Extraocular muscles revealed a right abduction restriction of -1 with the patient complaining of binocular horizontal diplopia at the right gaze, consistent with right abducens nerve palsy. Systemic neurological findings were normal, and imaging results were unremarkable. Diagnosis of right abducens nerve palsy post-dural puncture was made clinically. The patient was keen on conservative management instead of blood patch therapy. Hence, she was treated supportively via uni-ocular patching to relieve diplopia. Spontaneous complete recovery of the right abducens nerve palsy was observed after three weeks.

Cranial nerve palsy is a rare complication reported following spinal anesthesia, with the abducens nerve being the commonest nerve involved. Although it is not always benign, the presented case showed spontaneous complete recovery of the right abducens nerve palsy after three weeks. Awareness of this uncommon complication will avoid unnecessary distress and investigative burden to both the patient and the doctor.

## Introduction

Spinal anesthesia is a common procedure performed in obstetrics surgery. It is a relatively safe procedure with the experienced hand. Nevertheless, this procedure still has risks of complications, which encompassed post-dural puncture headache (PDPH), lumbar pain, hypotension, bradycardia, and epidural hematoma. Although it is rare, extraocular muscle paralysis may develop following a dural puncture, with the abducens nerve being the most common nerve involved [1]. This case report describes a case of isolated abducens nerve palsy following spinal anesthesia.

## Case presentation

A healthy 28-year-old lady, para 1, presented to the emergency department with persistent frontal headache, nausea, and vomiting following an emergency cesarean section four days ago. She had no issues antenatally. Unfortunately, she experienced difficulties with spinal anesthesia intrapartum. A 25-gauge Whitacre needle was utilized for administering spinal anesthesia under a sitting position. While inserting the needle, the anesthetist detected a loss of resistance, but upon removing the stylet, the clear cerebrospinal fluid was only of negligible amount. Six failed attempts were done before switching to general anesthesia. The patient underwent an emergency cesarean section due to fetal distress. She was not in labor during the attempts of spinal anesthesia. Otherwise, the cesarean section lasted for an hour and was uneventful. No intrapartum eclampsia or pre-eclampsia.

Postoperatively, the patient developed a mild frontal headache, which deteriorated on the fourth day. The headache was throbbing in nature and relieved by lying down but aggravated upon postural change from lying to an upright position. She also experienced nausea, vomiting, and giddiness but did not exhibit fever, neck pain, or limb weakness. During the examination, the patient's vital signs were stable, and blood tests, including a full blood count and renal profile, showed no abnormalities. She was diagnosed with post-dural puncture headache (PDPH) and received intravenous hydration, oral caffeine, and non-steroidal anti-inflammatory drugs (NSAIDs) which led to an improvement in her condition.

**Figure 1:**
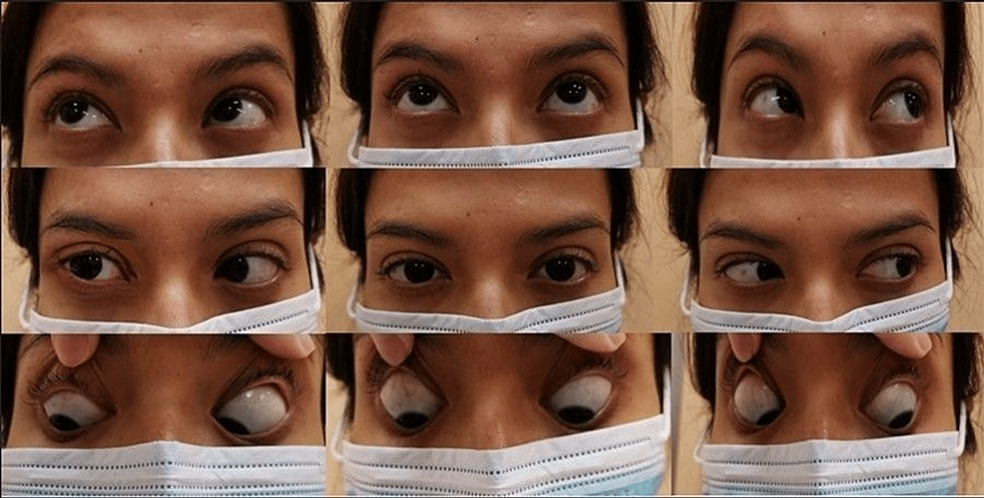
Right lateral rectus muscle restriction of -1 at right gaze

However, on the sixth day after the spinal anesthesia, she suddenly developed double vision, which was most prominent at the right gaze. Otherwise, she had no painful eye movement, swelling, blurring of vision, eye pain, or redness. Upon examination, her bilateral visual acuity was measured at 6/7.5, and there were no signs of proptosis or ptosis. The relative afferent pupillary defect was negative, with no anisocoria. Both eyes appeared orthophoric, with normal head posture. Extraocular muscles revealed a right abduction restriction of -1 with the patient complaining of binocular horizontal diplopia at the right gaze (Figure 1), consistent with right abducens nerve palsy. Otherwise, the anterior segment, tonometry, posterior segment, and systemic neurological findings were all unremarkable.

A clinical diagnosis of right abducens nerve palsy following a dural puncture was established. However, computed tomography of the brain was done to eliminate the potential risk of a life-threatening condition such as intracranial bleeding. The scan was reported to be normal. After receiving counseling, the patient pursued conservative management instead of undergoing a blood patch procedure. Therefore, she received supportive treatment through uni-ocular patching to alleviate diplopia, along with a continuation of medical treatment. She was discharged from the hospital on the eighth day, once her PDPH symptoms had completely resolved. Spontaneous complete recovery of the right abducens nerve palsy was observed after three weeks.

## Discussion

Cranial nerve palsy is a rare complication following dural puncture which may refer to an ophthalmologist or neurologist for intensive investigations. The reported incidence of cranial nerve palsies following dural puncture varies, ranging from 1 in 300 to 1 in 8000 cases. It is mostly reported after spinal anesthesia (47%), followed by myelography (18%), diagnostic lumbar puncture (12%), epidural anesthesia (11%), continuous spinal anesthesia (4%), and other dural puncture procedures (9%) [1-4].

During dural puncture, excessive leakage of cerebrospinal fluid (CSF) through the injection site can cause intracranial hypotension (ICH) when the CSF leakage is greater than the CSF production. In cases of ICH, the brain descends caudally, exerting traction forces on nearby cranial nerves that anchor the brain within the skull. The abducens nerve (83%) is the most affected, followed by the oculomotor nerve (14%) and the trochlear nerve (7%). These nerves can be affected by stretching or local compression. The abducens nerve is particularly vulnerable due to its long intracranial course, with three acute angulations between the dural entrance and its connection with the sympathetic plexus.

The caliber and type of spinal needle used for spinal anesthesia are the two main factors that affect the risk of post-dural puncture headache (PDPH). Quincke needles are specifically created to cut through dural fibers. On the contrary, Whitacre and Sprotte needles are blunt and intended to separate dural fibers. Shaikh et al. reported the incidence of PDPH after using 25G Quincke, 27G Quincke, and 27G Whitacre spinal needles was 8.3%, 3.8%, and 2.0% correspondingly [5]. By using a 25-gauge or smaller "atraumatic" needle, the incidence of PDPH is lowered to less than 1% [6,7]. Previous studies have shown that the majority of abducens nerve palsies occur with a needle size of 22 gauge or larger. The size of the needle used for dural puncture has been associated with the amount of cerebrospinal fluid leakage and the incidence of ICH. The use of a larger needle size, which creates a wider opening at the dural puncture site, is linked to an increased likelihood of cerebrospinal fluid (CSF) leakage. In the presented case, although a relatively smaller Whitacre needle size of 25 gauge was used, the multiple dural puncture attempts posed a higher risk of ICH.

The diagnosis of abducens nerve palsy resulting from intracranial hypotension is established through a process of excluding other potential causes. The differential diagnosis included neoplasm, ischemia, aneurysm, encephalitis, trauma, myasthenia gravis, and multiple sclerosis. Magnetic resonance imaging (MRI) of the brain is superior to computed tomography (CT) in ruling out intracranial inflammation or mass. Common radiographical signs include diffuse pachymeningeal enhancement with downward displacement of the brainstem, although these findings are not specific [3].

Therefore, clinical correlation is imperative in achieving the diagnosis. In our case, a CT brain was performed instead of an MRI due to the urgency of the situation. The MRI brain was unattainable at that moment urgently. The CT scan of the patient's brain revealed no abnormalities. Nishio et al. reported the window period ranging from day 1 to 3 weeks after dural puncture, with most cases developed at day 4-10 following dural puncture [8]. It is also worth noting that while multiple cranial nerve palsies may coexist in some cases, approximately 80% of cases are unilateral. This is consistent with the findings in our case.

We opted for a "wait-and-see" strategy and scheduled an MRI scan of the brain in the event of any new neurological symptoms or if the abducens nerve palsy persisted beyond three months. Fortunately, the abducens nerve palsy resolved on its own three weeks after the intracerebral hemorrhage resolved, and the patient made a full recovery without any lingering neurological deficits.

Abducens cranial nerve palsy following dural puncture is a favorable condition that carries a good prognosis, as approximately 80% of patients experience spontaneous resolution. Duffy and Crosby have reported that two-thirds of patients show complete resolution within one week, 25% remained symptomatic for over a month, and 10% persisted for more than three months [9].

For the last six decades, the epidural blood patch (EBP) has been regarded as the preferred and most effective method for treating PDPH, introduced by Gormley in 1960 [7]. Prompt EBP performed within 24 hours of cranial nerve palsy onset can lead to complete resolution of PDPH, with a success rate of 93% [10]. There is also emerging evidence suggesting that better outcomes are achieved when EBP is performed within 24 hours of cranial nerve palsy [11]. This is because by administering EBP, the duration of downward displacement and stretching of the abducens nerve is reduced, thus lowering the chances of persistent palsy [11,12]. However, administering an EBP more than 24 hours after the onset of cranial nerve (CN) palsy does not hasten the recovery of nerve function, even if it successfully relieves the associated headache [13-15]. In the presented case, the patient declined EBP within 24 hours of diagnosis. She was advised to use a uni-ocular eye patch for diplopia relief. Fortunately, the patient experienced full recovery after three weeks with spontaneous resolution.

Therefore, initial management of isolated abducens nerve palsy post-dural puncture without other neurological deficits should be observed for improvement given the majority of the reported cases having spontaneous resolution. Nevertheless, cranial nerve palsy following dural puncture that lasted for more than eight months was found to be permanent [16]. Johnson et al. reported the spontaneous recovery rate for unilateral isolated abducens nerve palsy is quoted as 78.4% with no improvement occurring after one year [17]. Hence, surgical correction of the squint may be appropriate if it persists for more than 12 months. Walsh suggested that consideration of corrective surgery on the extraocular muscle is advisable to be postponed with a lapse of at least 18 months because of protracted recovery [18]. In short, the treatment option for corrective surgery should be individualized by assessing the risks and benefits.

## Conclusions

Abducens nerve palsy following dural puncture is a rare complication that is less well-recognized by ophthalmologists when compared to anesthetists. This oversight often leads to costly investigations being conducted to rule out acute neurological conditions or tumors. Increasing awareness of this uncommon complication can help prevent unnecessary distress and minimize the investigative burden on the patient.
